# Baculoviral delivery of CRISPR/Cas9 facilitates efficient genome editing in human cells

**DOI:** 10.1371/journal.pone.0179514

**Published:** 2017-06-22

**Authors:** Sanne Hindriksen, Arne J. Bramer, My Anh Truong, Martijn J. M. Vromans, Jasmin B. Post, Ingrid Verlaan-Klink, Hugo J. Snippert, Susanne M. A. Lens, Michael A. Hadders

**Affiliations:** Center for Molecular Medicine, Section Molecular Cancer Research, University Medical Center Utrecht, Universiteitsweg 100, CG, Utrecht, The Netherlands; Wuhan Bioengineering Institute, CHINA

## Abstract

The CRISPR/Cas9 system is a highly effective tool for genome editing. Key to robust genome editing is the efficient delivery of the CRISPR/Cas9 machinery. Viral delivery systems are efficient vehicles for the transduction of foreign genes but commonly used viral vectors suffer from a limited capacity in the genetic information they can carry. Baculovirus however is capable of carrying large exogenous DNA fragments. Here we investigate the use of baculoviral vectors as a delivery vehicle for CRISPR/Cas9 based genome-editing tools. We demonstrate transduction of a panel of cell lines with Cas9 and an sgRNA sequence, which results in efficient knockout of all four targeted subunits of the chromosomal passenger complex (CPC). We further show that introduction of a homology directed repair template into the same CRISPR/Cas9 baculovirus facilitates introduction of specific point mutations and endogenous gene tags. Tagging of the CPC recruitment factor Haspin with the fluorescent reporter YFP allowed us to study its native localization as well as recruitment to the cohesin subunit Pds5B.

## Introduction

Recent advances in targeted genome engineering are revolutionizing biological research. Site specific targeting of nucleases such as zinc finger nucleases, transcription activator-like effector nucleases (TALENS) and the clustered regularly interspaced short palindromic repeats (CRISPR)/Cas9 system now allow genome editing in a wide variety of cultured cells as well as whole organisms. Of particular interest is the CRISPR/Cas9 system, due to its simplicity and ease of use [1–4]. The CRISPR/Cas9 system is based on the combination of a DNA endonuclease and a single guide RNA molecule (sgRNA) that directs the nuclease to a complementary target on the DNA where it induces double stranded breaks. In the majority of cases these lesions are repaired via non-homologous end joining (NHEJ) [5, 6]. This repair pathway is error-prone and as such can lead to indels that can cause frameshifts in the reading frame. When the size of the indel differs from a multiple of 3 nucleotides, transcription will result in nonsense mRNA and the use of an alternative stop codon. In this way, targeting Cas9 to coding regions gives rise to functional gene knockouts [7]. Alternatively, homology directed repair (HDR) can take place, in which case a homologous DNA template guides repair. The latter mechanism can be exploited to facilitate for example gene tagging or introduction of point mutations at endogenous loci by co-delivery of a repair template that harbors this specific feature [7].

Viral transduction serves as an efficient method for gene delivery, and can be employed for delivery of Cas9 or an sgRNA. Several common viral vectors have been used to deliver Cas9 and sgRNA expression cassettes into cells, including lentivirus, adenovirus and adeno-associated virus [2, 8–11]. However, all these systems suffer from a limited DNA carrying capacity due to constraints imposed by the size of the viral capsid. This poses a problem in the case of the relatively large gene encoding the commonly used *Streptococcus pyogenes* Cas9 (SpCas9), especially when used in combination with additional components such as the sgRNA expression cassette, selection markers or HDR templates. In such cases it is crucial that all components are delivered to the same target cells for maximal functionality.

Baculovirus is a well-established vector for gene delivery into a wide range of human cells with minimal cytotoxicity [12–17]. The commonly used baculovirus *Autographa californica* multiple nuclear polyhedrosis virus (AcMNPV) has a circular double stranded DNA genome of ± 134 kb. The virus can be manipulated in the form of a bacterial artificial chromosome (BAC). This allows easy insertion of foreign genes, under the control of mammalian control elements, into the baculovirus genome. Baculoviral transgene delivery is inherently transient in mammalian cells as baculovirus replication is restricted to insect cells and baculoviral DNA has a low integration frequency [16, 18]. Finally, baculoviral vectors have the capacity to harbor large segments of foreign DNA. Previous work has shown that baculoviral vectors can mediate expression of large genes including zinc finger nucleases, TALENS and Cas9 [19–21]. In fact, integration of fragments of up to 38 kb were stable and did not hamper the production of high titer virus [22]. We sought to harness the advantages described above and explore the use of baculovirus as a delivery system for Cas9 based genome-editing tools in human cells. As a proof of principle, we used the baculovirus system to genetically modify components of the chromosomal passenger complex (CPC) and one of its regulators, Haspin kinase. The CPC is a mitotic complex that consists of four proteins, named Borealin, INCENP, Survivin and Aurora B kinase [23]. During prometaphase and metaphase the CPC is enriched at inner centromeres. Concentration at the inner centromere is dependent on phosphorylation of histone H2A at threonine 120 by Bub1, and histone H3 phosphorylation at threonine 3 by Haspin [24–26]. During these stages of mitosis the CPC destabilizes erroneous kinetochore-microtubule attachments to promote chromosome bi-orientation, and it acts on the mitotic checkpoint, a surveillance mechanism that prevents anaphase onset until all chromosomes have been attached to the mitotic spindle. As such the CPC ensures faithful chromosome segregation [23]. Furthermore, in anaphase, the CPC translocates to the microtubules of the central spindle and promotes cytoplasmic division (cytokinesis) [23]. To disrupt the genes encoding the subunits of the CPC we developed a baculoviral transfer vector that combines SpCas9 and the sgRNA into a single vector. The resulting viruses had high transduction efficiencies in various human cell lines and resulted in a considerable proportion of cells with effective gene knockout. Furthermore, the incorporation of an HDR template into the same vector made it feasible to effectively introduce point mutations. Finally, we used baculoviral mediated delivery of Cas9 in combination with an HDR template to tag the CPC regulator Haspin, allowing us to study its native localization and recruitment by the cohesin component Pds5B.

## Results

### Generation of CRISPR/Cas9 baculoviruses

**Fig 1:**
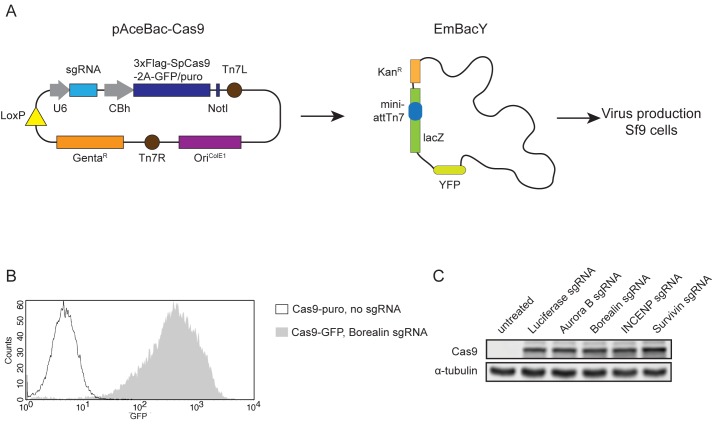
CRISPR/Cas9 baculovirus mediated Cas9 expression in U-2 OS cells. A) Schematic representation of the recombination of a pAceBac-Cas9 plasmid with a bacmid in EmBacY cells. The resulting bacmids were used for CRISPR/Cas9 baculovirus production in Sf9 cells. B) Representative FACS-profile showing GFP expression in U-2 OS cells treated with CRISPR/Cas9 baculovirus (MOI: 25). C) Western blot showing expression of Cas9 in U-2 OS cells treated with CRISPR/Cas9 baculoviruses (MOI: 25). α-tubulin was used as a loading control.

We set out to test the applicability of baculovirus as a delivery system for Cas9 based genome editing tools in a variety of commonly used cell lines. To this end we constructed a baculovirus transfer vector encoding 3xFLAG tagged SpCas9 fused to green fluorescent protein (GFP) with a 2A self cleaving sequence (Cas9-GFP) or 3xFLAG tagged SpCas9 fused to puromycin *N*-acetyl-transferase, again linked by a 2A self cleaving sequence (Cas9-puro), both under control of a CBh promoter. The transfer vector also contained an sgRNA expression module under control of the U6 promoter (Fig 1A). These vectors were used to produce baculoviruses that target the genes encoding the four members of the chromosomal passenger complex (CPC): *AURKB* (Aurora B sgRNA), *CDCA8* (Borealin sgRNA), *INCENP* (INCENP sgRNA) and *BIRC5* (Survivin sgRNA) with the goal of obtaining functional gene knockouts. We also created a virus that targeted the *Photynus pyralis LUCIFERASE* gene (Luciferase sgRNA) and a virus that did not contain an sgRNA sequence (no sgRNA), neither of which should cleave the specified target genes.

We first evaluated the performance of the Cas9 expressing baculoviruses in U-2 OS cells, a human osteosarcoma cell line that is highly susceptible to baculoviral transduction [27]. To ensure optimal infection, cells were transduced in RPMI-1640 medium, directly upon seeding [28, 29]. We examined GFP levels in transduced cells using flow cytometry since the amount of GFP should reflect Cas9 expression (Fig 1B and 1C). As a negative control, cells were transduced with Cas9-puro virus. Transduction of U-2 OS cells with various Cas9-GFP viruses resulted in close to 100 percent GFP positive cells and a high mean fluorescence intensity (Fig 1B and S1A Fig). Cas9 expression was further confirmed by Western blot analysis (Fig 1C and S1B Fig).

### Baculovirus mediated delivery of CRISPR/Cas9 leads to efficient gene knockout in U-2 OS cells

**Fig 2:**
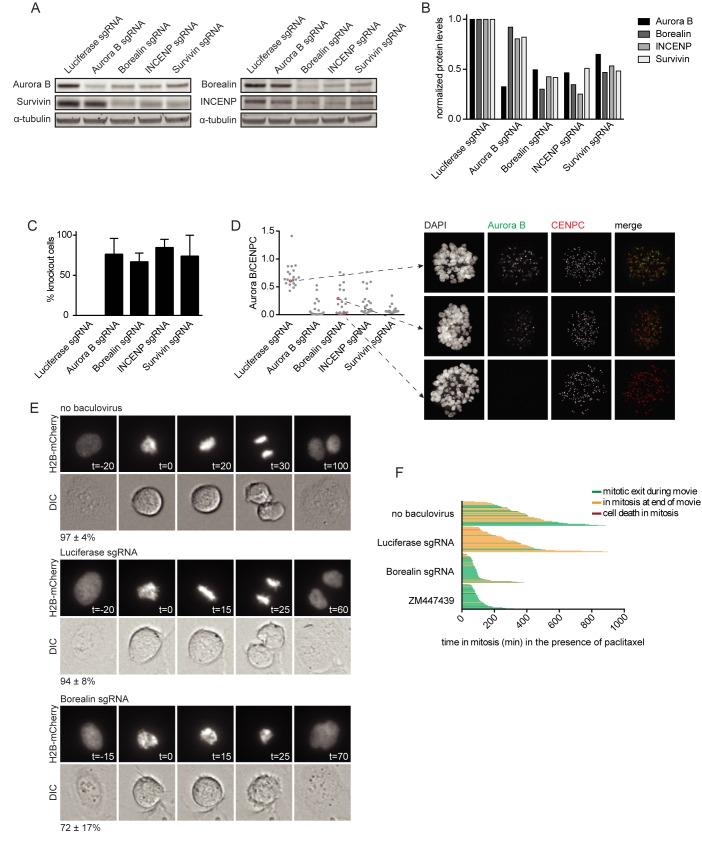
CRISPR/Cas9 baculovirus mediated knockout of CPC subunits in U-2 OS cells. A) Western blot of CPC members from mitotic U-2 OS cells treated with CRISPR/Cas9 baculoviruses (MOI: 25). α-tubulin was used as a loading control. B) Quantification of the Western blot shown in A. Protein levels were normalized over α-tubulin. C) Immunofluorescence images of mitotic U-2 OS cells treated with CRISPR/Cas9 baculoviruses (MOI: 25) were scored for loss of centromeric Aurora B. Bars represent the mean ± the standard deviation (SD) of 3 independent experiments. 100 cells were analyzed per experiment. D) Quantification of centromeric Aurora B levels in immunofluorescence images of mitotic U-2 OS cells treated with CRISPR/Cas9 baculoviruses (MOI: 25). Aurora B levels were normalized over CENPC. Thirty cells were analyzed per condition. Immunofluorescence images of the indicated cells are shown. E) Mitotic progression of H2B-mCherry U-2 OS cells that were transduced with the indicated CRISPR/Cas9 baculovirus (MOI: 25) and control cells. The timing of each frame is indicated in minutes, with the first frame in prometaphase set to t = 0. The average percentage of cells displaying the depicted phenotype and the SD are indicated. The numbers represent the average of 2 experiments and at least 10 cells were analyzed per condition for each experiment. F) Time in mitosis for U-2 OS cells that were transduced with the indicated CRISPR/Cas9 baculovirus (MOI: 25) in the presence of 1 μM paclitaxel. Cells treated with 2 μM ZM447439 were used as a positive control for the override of a paclitaxel-induced mitotic delay. Each bar represents a single cell. At least 48 cells were analyzed for each condition. The color of the bar indicates cell fate as depicted in the figure legend.

We next tested whether transduction of U-2 OS cells with CRISPR/Cas9 baculoviruses resulted in the disruption of the *AURKB*, *CDCA8*, *INCENP* and *BIRC5* loci. Cells were transduced with CRISPR/Cas9 baculoviruses and harvested after 48 hours, followed by isolation of genomic DNA. Genomic target sites were amplified by PCR and subjected to Sanger sequencing. The appearance of composite sequence traces after the predicted break sites confirmed effective cleavage and error-prone repair (S1C Fig). To obtain a more quantitative measure for the genome editing efficacy we analyzed the sequence data by Tracking of Indels by Decomposition (TIDE) analysis, which estimates the frequency of genome editing based on decomposition of the sequence trace [30]. This method revealed that the percentage of indels ranged between 60.1 and 89.3 for the different target loci (S1C Fig). We continued by testing whether the CRISPR/Cas9 baculovirus induced indels resulted in reduced levels of the corresponding protein. The protein levels of the CPC subunits are cell cycle regulated and peak in G2/mitosis [31–35]. Cells were therefore blocked in mitosis, 40 hours after transduction. The mitotic cell population was then harvested by shake-off and subjected to Western blot analysis. Viral transduction resulted in a reduction of target protein levels between 52 and 75 percent (Fig 2A and 2B, S2 Fig). Notably, Aurora B kinase knockout hardly affected protein levels of the non-enzymatic CPC subunits Borealin, INCENP, and Survivin (Fig 2A and 2B). This is in contrast with previous studies that reported a reduction of Survivin, INCENP, and to a lesser extent Borealin, upon knockdown of Aurora B using RNAi [34, 36]. Moreover, we observed that gene disruption and subsequent protein loss of either Borealin, INCENP or Survivin caused destabilization of all other CPC subunits (Fig 2A and 2B). The protein destabilizing effect of INCENP and Survivin knockout on Borealin and Aurora B was consistent with previous data obtained by siRNA or shRNA mediated knockdown of INCENP and Survivin [33, 34, 36], yet the effect of Borealin knockout seems to be different. Borealin siRNA mediated knockdown was shown to reduce Survivin protein levels, but Aurora B levels appeared unaffected in these studies. In addition, Borealin siRNA mediated knockdown reduced INCENP levels in one study but not in another one [31, 36].

To assess in more detail the proportion of cells in which treatment with Cas9 expressing baculovirus resulted in loss of protein, prometaphase cells were analyzed by immunofluorescence microscopy. Survivin and Borealin directly interact with the N-terminus of INCENP. This complex forms the centromere targeting module of the CPC and hence the presence of these subunits are not only required for Aurora B protein stability (Fig 2A) but also for inner centromere localization of Aurora B during (pro)metaphase [31, 37–40]. We therefore used the presence or absence of centromeric Aurora B as a read-out for the presence or absence of the CRISPR/Cas9 targeted chromosomal passenger proteins. We found that Aurora B was undetectable by eye in approximately 75% of mitotic U-2 OS cells, two days after transduction with Aurora B sgRNA, Borealin sgRNA, INCENP sgRNA or Survivin sgRNA expressing Cas9 baculoviruses (Fig 2C). Quantification of centromeric Aurora B levels revealed distinct cell populations with varying levels of Aurora B (Fig 2D and 2E). These included cells with Aurora B levels similar to the Luciferase control, and cells in which Aurora B was undetectable, indicating that no functional CPC gene was present. However, we also found cells in which Aurora B centromere levels were still detectable, but clearly reduced compared to control cells (Fig 2D and 2E). This intermediate pool most likely represents the cells with heterozygous disruption of the targeted gene.

Having established high knockout efficiency for all four CPC sgRNA expressing Cas9 baculoviruses, we continued with one of the CPC sgRNA (i.e. Borealin) expressing Cas9 baculoviruses to test whether knockout of this CPC target gene also resulted in loss of CPC function. The CPC is essential for proper chromosome alignment, mitotic checkpoint function and cytokinesis [23]. To analyze these processes we followed mitotic progression of U-2 OS cells, stably expressing H2B-mCherry, using live cell fluorescence microscopy. We started imaging cells two days after transduction and found that 72% ±17 of the cells treated with a Borealin sgRNA virus exited mitosis before proper chromosome alignment, and they failed cytokinesis (Fig 2F). Moreover, treatment with Borealin sgRNA virus resulted in an override of a mitotic checkpoint-dependent arrest induced by the microtubule stabilizing agent paclitaxel, similar to treatment with the Aurora B inhibitor ZM447439 (Fig 2G). These observations are in line with previously established effects of Aurora B inhibition or knockdown of CPC subunits [31, 33, 36, 39–43], confirming that the observed phenotypes can indeed be attributed to CPC disruption. Together, these experiments demonstrate that baculoviral transduction with Cas9/sgRNA expressing viruses leads to efficient functional gene knockout in U-2 OS cells. Moreover, since we already observed profound effects two days after virus transduction, the CRISPR/Cas9 baculoviruses could serve as a useful alternative for RNAi-mediated knockdown of at least short lived (cell cycle) proteins.

### Gene knockout by baculovirus mediated CRISPR/Cas9 delivery in a panel of cell lines

**Fig 3:**
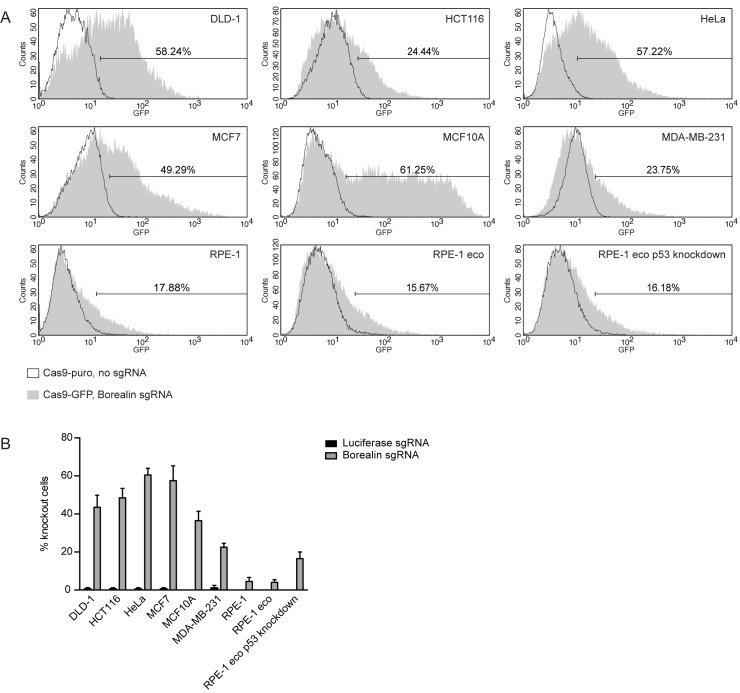
Effect of CRISPR/Cas9 baculoviral transduction in a panel of cell lines. A) Representative FACS-profiles showing GFP expression in cells treated with Cas9-GFP baculovirus (MOI: 75). The markers are set such that 2% of the cells treated with Cas9-puro baculovirus are included in this region. The percentage of cells treated with Cas9-GFP baculovirus within the marker region is indicated. B) Immunofluorescence images of mitotic cells treated with the indicated Cas9-GFP baculoviruses (MOI: 75) were scored by eye for the presence or absence of centromeric Aurora B. The bars represent the mean ± SD of 2 experiments. At least 24 cells were analyzed per experiment per condition.

U-2 OS cells are known to be highly susceptible to transduction with baculovirus [27] However, we were also interested in the applicability of Cas9 expressing baculoviruses in a broader range of cell lines. We selected a panel of 7 frequently used cell lines, including two colon cancer cell lines (DLD-1 and HCT116), one cervical cancer cell line (HeLa), two breast cancer cell lines (MCF7 and MDA-MB-231) and two non-transformed cell lines (MCF10A and RPE-1). To assess the efficiency of viral transduction we determined Cas9 linked GFP expression using flow cytometry following treatment with Borealin sgRNA virus and Cas9-puro virus expressing no sgRNA as a control (Fig 3A). The percentages of GFP positive cells ranged between 18 and 61, with the order of the GFP positive fraction being MCF10A > DLD-1 > HeLa > MCF7 > HCT116 > MDA-MB-231 > RPE-1 (Fig 3A). FACS analysis further showed that the levels of GFP expression varied between different cell lines as measured by the mean fluorescence intensity. For example, while the percentage of GFP positive cells is similar between MCF10A, DLD-1, and HeLa cells, MCF10A cells display the highest fluorescent signal per cell (Fig 3A). The variation in the percentage of GFP positive cells and mean fluorescence intensity between cell lines likely reflect cell type specific differences in the susceptibility to baculoviral transduction.

For each cell line, we again looked in more detail in what fraction of cells treatment with Cas9-GFP baculovirus resulted in loss of the targeted protein. Cells were treated for two days with baculovirus expressing Cas9-GFP Borealin sgRNA or Cas9-GFP Luciferase sgRNA and then blocked in prometaphase. We again analyzed centromeric localization of Aurora B as a read out for Borealin expression by immunofluorescence microscopy. The observed knockout scores ranged from 4 to 60 percent (Fig 3B). Since we assessed the absence or presence of centromeric Aurora B in the total cell population and did not select for GFP positive (i.e. Cas9 expressing) cells, we expected that the fraction of Aurora B negative (Borealin knockout) cells would be equal to or smaller than the percentage of GFP positive cells. For DLD-1, HeLa, MCF7, and MDA-MB-231 cells the knockout percentage was indeed within the range of the GFP+ fraction. However, the fraction of knockout cells did not always correlate with the percentage of transduced cells. In HCT116 cells the proportion of knockout cells exceeded the percentage of GFP positive cells as determined by flow cytometry (Fig 3A and 3B). Cells expressing very low amounts of Cas9-GFP overlap with the Cas9-puro control population and were hence not marked as GFP positive. As a consequence, the measured percentage of Cas9-GFP positive cells is likely an underestimation of the total number of cells infected and further suggests that very low levels of Cas9 expression are likely sufficient to generate knockouts. Conversely, in MCF10A cells and RPE-1 cells the percentage of knockout cells was relatively low compared to the fraction of GFP positive cells. Both cell lines are non-transformed and have functional p53. We hypothesized that CRISPR/Cas9 mediated introduction of double strand breaks would activate a p53-dependent DNA damage response in these cells and induce a G1 or G2 cell cycle arrest. This would then prevent the detection of knockout cells in the mitotic population. To test this possibility we transduced RPE-1 cells that stably expressed a short hairpin RNA against p53 (RPE-1 eco p53kd). As a control we used the parental, p53 positive cell line (RPE-1 eco). While both cell lines were transduced with similar efficiency as RPE-1 cells (Fig 3A), the percentage of observed knockout cells increased approximately 4 fold in the RPE-1 eco p53kd cell line as compared to RPE-1 or RPE-1 eco cells, in line with the amount of cells transduced (Fig 3B). In conclusion, CRISPR/Cas9 baculoviruses can be used to generate knockouts with relatively high efficiency in multiple cell lines, although the number of affected cells varies due to differences in virus transduction efficiency and specific cell line characteristics, such as p53 status.

### Introduction of point mutations using CRISPR/Cas9 baculovirus

**Fig 4:**
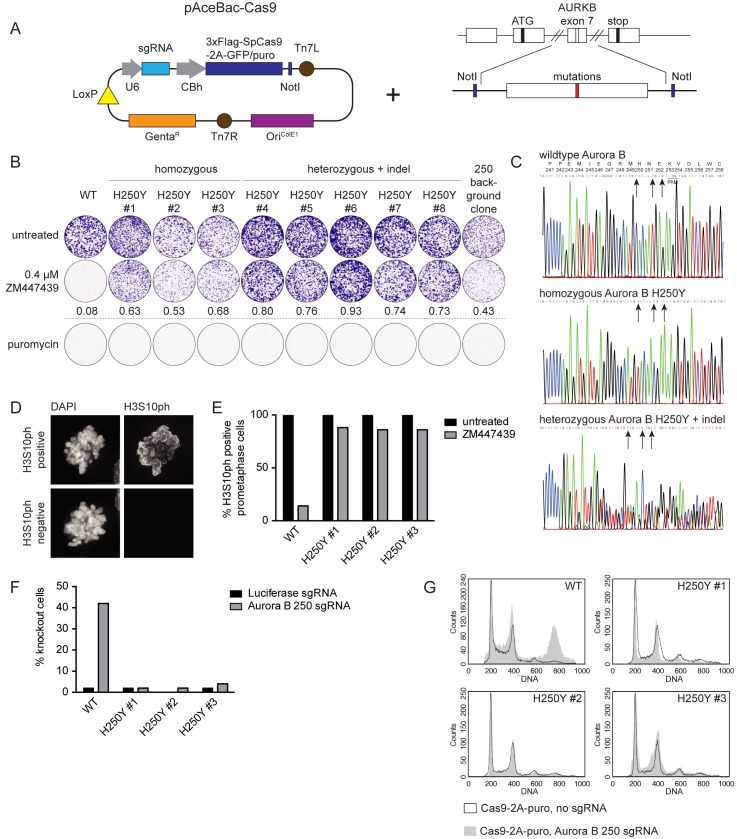
Introduction of the H250Y mutation in Aurora B. A) Schematic representation of the introduction of an HDR template carrying point mutations in the pAceBac-Cas9 plasmid. B) Colony formation of HCT116 cells in ZM447439 with cells carrying either wildtype Aurora B alleles, or alleles with homozygous H250Y mutations, or with a heterozygous mutation for H250Y combined with an indel in the other allele. The numbers indicate colony outgrowth in the ZM447439 treated conditions relative to the untreated conditions. Clones were also tested for their capacity to form colonies in the presence of puromycin. C) Sequence chromatograms of the Aurora B locus in the vicinity of H250. Depicted is the Aurora B sequence trace of wildtype HCT116 cells or clones carrying either the homozygous Aurora B H250Y mutation or the heterozygous Aurora B H250Y mutation combined with an indel. The PAM site is indicated and the arrows point out the base substitutions that were introduced. The corresponding amino acid sequence is shown in the top graph. D) Representative immunofluorescence images of prometaphase cells that have either retained or lost histone H3 Serine 10 (H3S10) phosphorylation after treatment with ZM447439 (0.5 μM). E) Quantification of immunofluorescence images of prometaphase cells depicted in (D) Cells were scored for H3S10ph loss following treatment with ZM447439 (0.5 μM). F) Immunofluorescence images of wildtype HCT116 cells and clones harboring homozygous Aurora B H250Y mutations treated with the indicated CRISPR/Cas9 baculoviruses (MOI: 75) were arrested in mitosis and scored for loss of centromeric Aurora B. G) Representative FACS-profiles showing the DNA content of wildtype HCT116 cells and clones harboring homozygous Aurora B H250Y mutations that were treated with the indicated CRISPR/Cas9 baculoviruses (MOI: 75). Puromycin treatment was used to select for transduced cells and samples were harvested 4 days after transduction.

To investigate the usability of baculovirus as a vector for generating specific genome alterations we added a ~300 base pair HDR template to the Cas9-puro plasmid (Fig 4A). We selected Aurora B as our target, specifically the H250Y mutation. This amino acid change was previously shown to confer resistance against the Aurora B inhibitor ZM447439 in HCT116 cells [44]. We chose an sgRNA sequence that targets Cas9 to a locus in exon 7 that is in close proximity to the DNA site encoding Aurora B amino acid 250 (Aurora B 250 sgRNA) and designed the HDR template to introduce the Aurora B H250Y mutation together with two additional silent mutations to prevent cleavage by Cas9 once the mutation has been introduced (H250Y template). HCT116 cells were transduced with viruses containing Cas9-puro plus the Aurora B 250 sgRNA and H250Y template, the Aurora B 250 sgRNA alone, or no sgRNA. We then selected with puromycin for 16 hours to enrich for transduced cells, followed by the addition of ZM447439 to select for cells that had successfully acquired the Aurora B H250Y mutation. From the cell population treated with Aurora B 250 sgRNA + H250Y template, 8 clones grew out, whereas 1 clone survived in the Aurora B 250 sgRNA treated control population. No clones grew out after treatment with a virus expressing Cas9-puro but lacking an sgRNA. Of the 8 selected clones, 3 were homozygous for the H250Y mutation, whereas the other 5 had a monoallelic introduction of the desired point mutation in combination with an indel in the second allele (Fig 4B and 4C). No additional mutations were found in the *AURKB* coding region in any of the obtained clones, including the single clone expanded from the Aurora B 250 sgRNA treated control cells, which did however have a heterozygous indel at the sgRNA target site. Resistance of the clones to Aurora B inhibition was confirmed by their colony forming capacity in the presence of ZM447439 (Fig 4B). Furthermore, analysis of histone H3 Serine 10 phosphorylation, a known substrate of Aurora B [42, 45], confirmed that the Aurora B H250Y mutant was active even in the presence of ZM447439 (Fig 4D and 4E). At the same time, all clones had regained sensitivity to puromycin (Fig 4B), demonstrating that Cas9-puro had not integrated into the genome.

The introduction of three mutations in the HDR template should prevent Cas9-mediated cleavage of both the transduced template and of the genomic target locus after successful introduction of these mutations. Cell lines resistant to Cas9-mediated editing of a particular target gene could serve as a control in verifying the specificity of an observed phenotype upon Cas9 mediated knockout. This strategy of introducing endogenous (silent) mutations has recently been used to verify siRNA specificity [46]. To validate this approach for Cas9-mediated knockout we investigated whether the three nucleotide substitutions were indeed sufficient to prevent further CRISPR/Cas9 activity towards this site. Treatment of wildtype HCT116 cells with the Aurora B 250 sgRNA virus resulted in Aurora B knockout in 42 percent of the cells in mitosis based on immunofluorescence staining (Fig 4F). This is similar to the knockout efficiency of Borealin in this cell line (Fig 3B). In contrast, Aurora B centromere localization remained unaffected by the Aurora B 250 sgRNA virus in the 3 homozygous Aurora B H250Y clones (Fig 4F). Furthermore, the homozygous Aurora B H250Y clones were resistant to polyploidization resulting from treatment with Aurora B 250 sgRNA virus and concomitant Aurora B knockout (Fig 4G). These data demonstrate that introduction of mutations at the Cas9/sgRNA target site is a feasible approach to create cell lines that can be used to validate Cas9-mediated knockout phenotypes.

### Endogenous tagging of Haspin using CRISPR/Cas9 baculovirus

**Fig 5:**
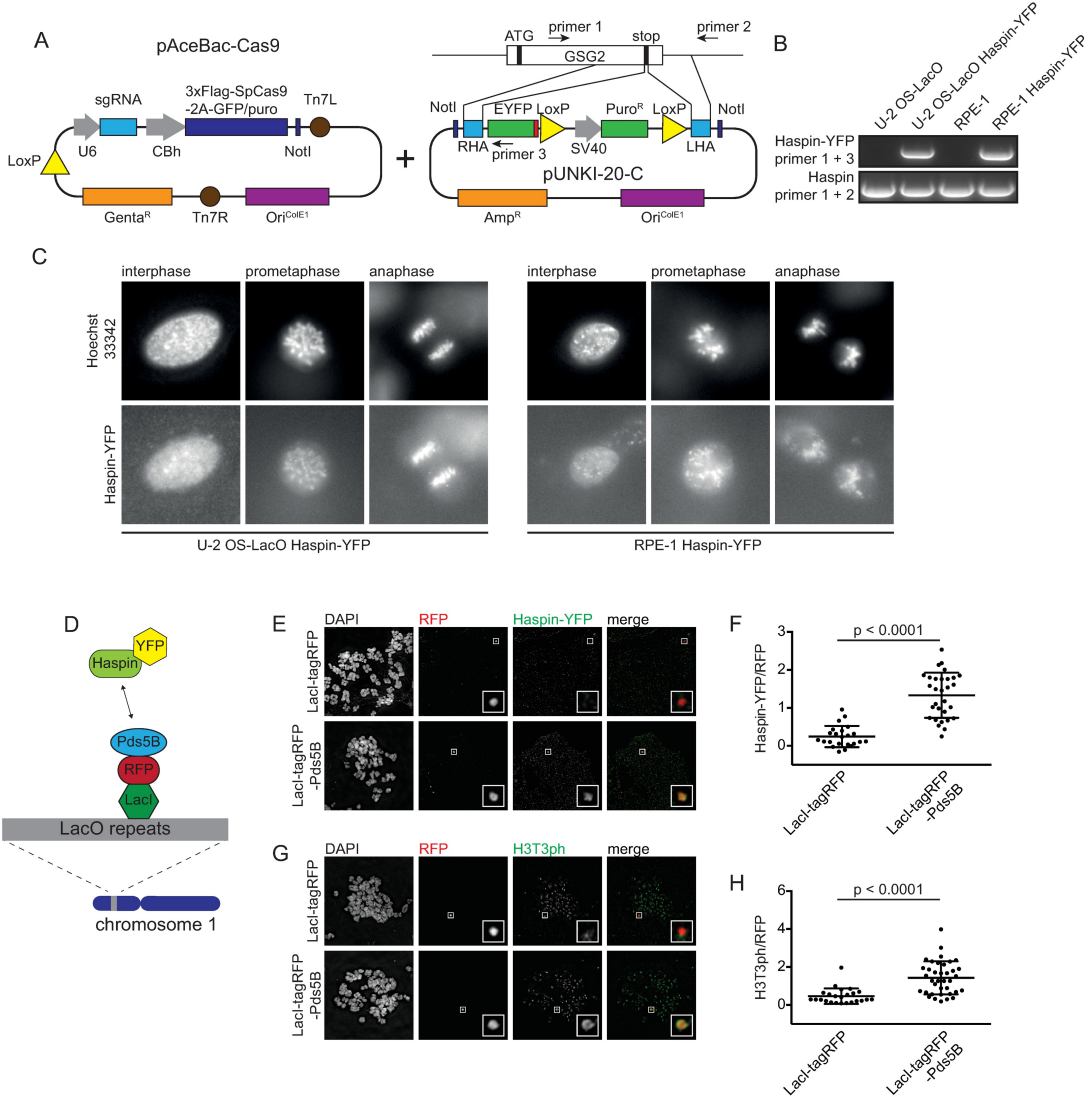
Endogenous tagging of Haspin using CRISPR/Cas9 baculovirus. A) Schematic representation of the introduction of an HDR template for endogenous tagging of *GSG2* in the pAceBac-Cas9 plasmid. B) Integration of the YFP-tag at the C-terminus of the gene encoding Haspin was confirmed by PCR using the indicated primer pairs, schematically depicted in Fig 5A. The PCR product obtained using primers 1 and 2 is of the untagged allele, based on its size. C) Representative live cell images of U-2 OS-LacO Haspin-YFP cells and RPE-1 Haspin-YFP cells in prometaphase and interphase. D) Schematic overview depicting the binding of LacI-tagRFP-Pds5B to the LacO repeats. Upon interaction between Pds5B and Haspin an YFP signal can be detected at this ectopic locus. E) Immunofluorescence images of metaphase spreads of U-2 OS-LacO Haspin-YFP cells expressing LacI-tagRFP or LacI-tagRFP-Pds5B and stained for DAPI, YFP and RFP. F) Quantification of Haspin-YFP at the LacO locus. Depicted is the mean of Haspin-YFP normalized over RFP ± SD. Each dot represents a single cell. The data was analyzed using an un-paired Student’s t-test. G) Immunofluorescence images of metaphase spreads of U-2 OS-LacO Haspin-YFP cells expressing LacI-tagRFP or LacI-tagRFP-Pds5B stained for DAPI, H3T3ph and RFP. H) Quantifications of H3T3ph at the LacO locus. Depicted is the mean of H3T3ph levels normalized over RFP ± the SD. Each dot represents a single cell. The data was analyzed using an un-paired Student’s t-test. A minimum of 23 cells was analyzed per experiment.

The kinase Haspin contributes to inner centromere localization of the CPC via phosphorylation of histone H3 at threonine 3 (H3T3ph), which forms a docking site for the CPC subunit Survivin [24–26]. While CPC localization has been extensively studied, relatively little is known about how Haspin is recruited to chromatin and how its localization is regulated, in part due to a lack of tools that allow visualization of the endogenous protein [47, 48]. We therefore chose to tag endogenous Haspin with the fluorescent reporter yellow fluorescent protein (YFP). Since baculovirus can accommodate large fragments of foreign DNA we set out to design a single virus format for the tagging of endogenous genes. To this end we constructed a HDR plasmid that contains the coding sequence of YFP and a puromycin resistance cassette flanked by restriction sites that can be used to introduce homology arms (Fig 5A). The HDR template was subsequently introduced into the Cas9-GFP baculovirus donor plasmid for the construction of a single virus containing all elements required for genome editing (Fig 5A). For tagging of the Haspin encoding gene *GSG2* we selected an sgRNA sequence targeting the 3’ end of the gene and cloned homology arms of approximately 1 kb in length corresponding to the sequence adjacent to the stop codon (Fig 5A). We used the resulting virus to transduce U-2 OS cells containing an integrated LacO array (U-2 OS-LacO) [49] and RPE-1 cells, followed, after 48 hours, by puromycin selection. Despite the relatively low transduction and knockout efficiency in RPE-1 cells (see Fig 3A and 3B), we were able to obtain several puromycin resistant RPE-1 clones after transduction with the baculovirus for *GSG2* tagging. YFP integration was confirmed by PCR analysis (Fig 5B) and sequencing. PCR analysis further revealed that all clones had heterozygous integration of the tag. We next set out to examine the localization of Haspin-YFP. While we were unable to visualize the protein by immunofluorescence of fixed cells, live cell imaging revealed that Haspin-YFP localized to the nucleus in interphase cells (Fig 5C). Notably, during live cell imaging the Haspin-YFP signal was very weak, indicating that the protein is likely of low abundance. In mitosis, Haspin-YFP was observed to be dispersed over the chromatin (Fig 5C and S3 Fig). Although previous studies have reported accumulation of overexpressed GFP-Haspin on centromeres [47], we were unable to discern sites of Haspin-YFP enrichment on the DNA. This is likely due to the faint signal and the limited resolution as a consequence of detector binning. Interestingly, we do not observe a decrease in Haspin-YFP during anaphase, when cohesin is removed from the chromatin (Fig 5C). It has been suggested that Haspin is recruited to chromatin by the cohesin-associated protein Pds5B [26, 50]. To test whether Pds5B can indeed recruit Haspin we made use of U-2 OS-LacO cells expressing endogenously tagged Haspin-YFP. These cells harbor an array of approximately 200 times 256 LacO repeats on chromosome 1 [49]. The LacO array allows specific recruitment of LacI fusion proteins to this ectopic locus that can be visualized by immunofluorescence microscopy. We fused Pds5B to LacI-tagRFP and expressed the fusion protein in U-2 OS-LacO Haspin-YFP cells through baculoviral transduction (Fig 5D). Cells expressing either LacI-tagRFP-Pds5B or LacI-tagRFP as a control were blocked in mitosis and chromosome spreads were analyzed. Spreads were stained with antibodies against GFP to visualize Haspin-YFP, RFP to visualize LacI-tagRFP-Pds5B and H3T3ph as a read-out for Haspin kinase activity. We found that Haspin-YFP is clearly enriched on the LacO array in the presence of LacI-tagRFP-Pds5B in contrast to LacI-tagRFP alone (Fig 5E and 5F). Furthermore, targeting LacI-tagRFP-Pds5B to LacO arrays resulted in histone H3 phosphorylation on threonine 3, indicating that the recruited Haspin was indeed active (Fig 5G and 5H).

## Discussion

For over thirty years the baculovirus expression vector system has had a profound impact on basic research as a method for simple, high-level production of recombinant proteins in insect cells [51]. The subsequent discovery that baculovirus is also capable of infecting mammalian cells has resulted in numerous novel applications ranging from basic cell biology tools to potential gene therapy vectors. The latter is of particular interest due to the recent explosion of activity in the development of genome editing technologies, particularly those based on the CRISPR/Cas9 system. Here, we demonstrate the use of baculovirus as a delivery vehicle for Cas9 based genome-editing tools in multiple human cell lines.

Viral transduction serves as an efficient method for gene delivery, and can be employed for delivery of CRISPR components. The use of a lentiviral vector that combines Cas9 and an sgRNA has previously been described [8]. However, viral titers are reduced approximately 100 fold due to the size of the CRISPR components. Furthermore, lentiviral expression results in stable integration of the CRISPR components in the host genome. This could cause unwanted gene disruption as a consequence of genomic insertion. Moreover, constitutive expression of Cas9 is inherently associated with the ongoing risk of acquiring off-target cleavages [52]. Adeno-associated viruses (AAV) have a predominantly episomal nature, with low frequency integration at a specific locus [53] and several research groups have investigated the use of AAV as a vehicle for Cas9 and sgRNA delivery [2, 9, 54]. Again, a major drawback is the limited packaging capacity of AAV vectors [55]. This problem has been addressed in part by the use of smaller Cas9 homologues or adopting a co-expression strategy using separate AAV vectors for Cas9 and the sgRNA, or by making use of trans-splicing, whereby two parts of Cas9 expressed from separate AAV vectors are spliced together in cells to form a functional enzyme [9, 54]. Finally, adenoviral vectors, which lack the machinery required for integration into the host genome [56] and have a relatively large cloning capacity [55], have been demonstrated to be efficient at mediating Cas9 and sgRNA delivery and induce site-specific double strand breaks in cultured cells and in vivo [10, 11, 57, 58]. However, the use of adenovirus requires handling in a biosafety level 2 environment. The use of baculovirus as a vehicle for CRISPR/Cas9 machinery is a compelling alternative, since it is able to transduce a wide range of mammalian cells [17, 19, 20]. Moreover, baculoviruses do not integrate into the host genome and are not known to cause disease in humans [18]. Its ability to carry large amounts (<38 kB) of foreign DNA makes it ideally suited not only to deliver Cas9 and sgRNA sequences but also allows the inclusion of multiple additional elements such as selection markers, fluorescent probes and HDR templates, all in a single virus. This ensures infection always results in co-delivery of all required components into the target cell.

We constructed a baculoviral vector that expresses both Cas9-2A-GFP and an sgRNA sequence. Using CRISPR/Cas9 baculovirus we were able to efficiently transduce a panel of cell lines. Furthermore, transduction resulted in fast and efficient gene knockout of all four members of the CPC. This suggests this method can also be used in transient depletion experiments analogous to siRNA-mediated knockdown. As is the case for siRNA experiments the success of complete protein depletion will be largely dependent on the rate of protein turnover. We found that knockout of any one of the three non-enzymatic subunits of the CPC, Borealin, INCENP and Survivin, resulted in destabilization of all other members of the complex. This is in line with several observations that show a strong functional interdependence between the four CPC subunits that seem to be centered on INCENP. An N-terminal region in INCENP, called the CEN-box, forms a tripartite coiled coil with Borealin and Survivin and the in vitro interaction between these subunits occurs in the absence of Aurora B [37]. The C-terminus of INCENP, called the IN-box, interacts with Aurora B, thereby linking it to the rest of the complex [31, 37, 38, 59]. Moreover, Aurora B stability has also been shown in vitro to depend on its interaction with INCENP [60]. Combined with our observations in the knockout cells (Fig 2A and 2B), we favor a model in which Borealin, INCENP and Survivin form a stable core complex independently of Aurora B, while Aurora B protein stability requires its interaction with this complex.

We observed that the rate of functional gene knockout largely correlates with the transduction efficiency. Comparing knockout efficacy with other viral delivery systems however is difficult due to the large number of variables including different target genes, different sgRNA sequences, different cell types and different strategies to measure actual efficacy. For example, lentiviral expression of Cas9 + sgRNA resulted in efficient knockout of an EGFP reporter in HEK293 cells [8]. However, efficacy was assessed after 11 days of puromycin selection based on the expression of the stably integrated Cas9-2A-puromycin *N*-acetyl-transferase and thus cannot be directly compared. Maggio et al. have compared adenoviral vectors (co-infection of Cas9 and sgRNA expressing viruses) in multiple cell lines and showed 31% and 18% target gene (the *AAVS1* locus) knockout frequency for HeLa cells and U-2 OS cells respectively [11], compared to ± 60% and 65% as observed by us using baculoviral delivery of Cas9 and targeting the *CDCA8* locus (Fig 2C for U-2 OS cells and Fig 3B for HeLa cells). Ultimately, we think that since absolute efficacy is rarely described in a consistent manner, due in part to the many variables involved, comparisons are difficult to make. We argue that for most cell lines we tested, the efficacy is mostly determined by the transduction rate, as the percentage of knockouts is typically comparable to the percentage of cells transduced.

For viral transduction we employed a generic protocol that works well for many commonly used cell lines. However, baculoviral transduction efficiency is cell line specific and subject to many variables, including the multiplicity of infection (MOI), the type of medium and the time and temperature of transduction [28, 29, 61]. Furthermore, viral pseudotyping, histone deacetylase inhibitors and the use of post-transcriptional regulatory elements or different promoters have al been shown to influence the level of transduction efficiency or transgene expression [15, 62, 63]. Optimization of these variables will allow researchers to achieve optimal expression levels in the system of their choice. While knockout efficiency largely correlated with viral transduction levels, we did observe that non-transformed RPE-1 cells yielded very few knockouts in the mitotic population, even though a substantial part of the population expressed CRISPR/Cas9. Stable knockdown of p53 resulted in an increase of knockout cells in line with the number of cells displaying Cas9 expression, suggesting that p53 prevents successfully targeted cells from entering mitosis, most likely through induction of a cell cycle arrest or apoptosis. This could further explain why non-transformed MCF10A cells also show relatively low knockout rates compared to other cell lines, despite expressing much higher levels of Cas9 [64, 65]. Thus, transient depletion of p53 together with CRISPR/Cas9 baculovirus treatment may form an effective solution in increasing efficiency in non-transformed cells as this would by-pass the p53-associated selection against knockout cells. Nonetheless, we were able to successfully tag endogenous Haspin in RPE-1 cells without depleting p53.

Cas9 mediated cleavage of DNA is typically repaired, either through NHEJ or HDR. The latter repair mechanism can be exploited to locally introduce novel features, such as point mutations or fluorescent reporters, when offering an appropriate donor sequence. Endogenous tagging allows the examination of proteins without depending on overexpression or on the availability of antibodies while the introduction of, for example disease associated mutations, allows the study of phenotypes in a setting not dependent on many of the drawbacks associated with overexpression knockdown/add back studies such as variation in expression levels and knockdown efficiency. We used CRISPR/Cas9 baculovirus to introduce specific point mutations in Aurora B and tag the kinase Haspin with the fluorescent reporter YFP. In both cases the HDR template could be included in the same virus owing to its large capacity for accepting foreign DNA. Moreover, the large capacity further allowed for the introduction of a puromycin resistance cassette in the 3’ UTR of Haspin, facilitating the identification of correct clones through antibiotic selection. However, despite efficient delivery of CRISPR/Cas9 components together with an HDR template into target cells, the limited contribution of HDR compared to NHEJ for double strand break repair remains a bottleneck for precise genome editing. Various strategies have been developed to shift the balance of DNA repair towards HDR through, for example: cell cycle synchronization, inhibition of NHEJ, via the ligase IV inhibitor SCR7 or the depletion of the NHEJ proteins KU70 and KU80 [66, 67], or alternatively, stimulation of the HDR protein RAD51 [68, 69]. For the endogenous tagging of Haspin, none of the aforementioned approaches were used, yet we were able to efficiently generate cell lines in which the gene was successfully tagged. However, direction of the DNA repair pathway towards HDR may be more critical in cases where it is not possible to select for the desired genomic alteration.

The kinase Haspin plays a critical role in regulating recruitment of the CPC to the inner centromere. While inner centromere localization is thought to be critical for the function of the CPC, little is known about how Haspin is regulated. Research regarding Haspin has been in part impeded by the difficulty of detecting the protein, and was therefore largely based on Haspin overexpression of tagged variants [47, 48]. Live cell fluorescent imaging of endogenously tagged Haspin-YFP cells, both in U-2 OS-LacO and RPE-1 cells, revealed that the signal of Haspin was weak. This suggests that Haspin may be expressed at very low levels. Nevertheless, we were able to image endogenous Haspin during the cell cycle, showing it is present in the nucleus during interphase and is associated with chromatin throughout mitosis. Furthermore, the U-2 OS-LacO Haspin-YFP cell line allowed us to study recruitment of Haspin to the cohesin-associated factor Pds5B. Interestingly, our data show that Haspin remains associated with mitotic chromatin even after anaphase onset. Anaphase onset is associated with the proteolytic removal of cohesin from the chromatin through activation of the protease separase. This implies that regulation of Haspin recruitment may be more complex than the proposed interaction with cohesin/Pds5B. Indeed, a recent study suggests that the chromatin association of Pds5B itself may be independent of the cohesin complex as knock out of the cohesin subunit Scc1 resulted in a loss of the core cohesin components SMC1, SMC3 and SA2 from the chromatin, but not of Pds5B [70]. It is likely that Pds5B retains its association with chromatin through its ability to directly bind to DNA via two C-terminal AT-hook domains [71]. Furthermore, two recent reports reveal a critical role for sumoylated TOP2A in the recruitment of Haspin to centromeres during mitosis [72, 73]. How TOP2A, sumoylation, cohesin and Pds5B cooperate to recruit Haspin to the inner centromere will require further investigation. Endogenously tagged Haspin-YFP cell lines will form a powerful tool to further study the regulation of Haspin.

Besides its role in research, the use of genome editing tools holds great potential for clinical applications. Plans to initiate the first trials using CRISPR/Cas9 for therapeutic purposes were recently announced [74]. Interestingly, baculoviruses have been suggested as suitable vehicles for gene therapy, because of their low cytotoxicity, low frequency of genomic integration and their inability to replicate in mammalian cells [75]. Baculovirus mediated delivery of CRISPR/Cas9 could form an important tool for genomic engineering of patient cells.

## Materials and methods

### Cell culture

U-2 OS (ATCC, HTB-96), HeLa (ATCC, CCL-2) and DLD-1 (a gift from Dr. Onno Kranenburg, UMC Utrecht, Utrecht, NL) cells were cultured in DMEM (Sigma-Aldrich, St. Louis, MO, cat. no. D6429) supplemented with 6% fetal bovine serum (FBS) (Sigma-Aldrich, St. Louis, MO, cat. no. F7524), 2 mM UltraGlutamine (Lonza, Basel, Switzerland, cat. no. BE17-605E/U1), 100 units/ml penicillin and 100 μg/ml streptomycin (Lonza, Basel, Switzerland, cat. no. DE17-602E), hereafter called DMEM. RPE-1 (ATCC, CRL-4000), RPE-1 eco, RPE-1 eco p53kd (both a gift from Dr. Roderick Beijersbergen, NKI, Amsterdam, NL), U-2 OS-LacO [49], MCF7, and MDA-MB-231 cells (both a gift from Dr. Patrick Derksen, UMC Utrecht, Utrecht, NL) were cultured in DMEM F12 (Lonza, Basel, Switzerland, cat. no. BE04-687F/U1) supplemented with 10% FBS, 2 mM UltraGlutamine and 100 units/ml penicillin and 100 μg/ml streptomycin, hereafter called DMEM F12. HCT116 cells (a gift from Dr. Onno Kranenburg) were cultured in DMEM supplemented with 10% FBS. MCF10A cells were cultured in DMEM F12 supplemented with 5% FBS, 5 ng/ml epidermal growth factor, 0.5 μg/ml hydrocortisone, 100 ng/ml cholera toxin, 5 μg/ml insulin, 100 units/ml penicillin and 100 μg/ml streptomycin with UltraGlutamine omitted. All human cell-lines were maintained at 37°C and 5% CO_2._ U-2 OS cells stably expressing H2B-mCherry were generated by lentiviral transduction. Sf9 cells (a gift from Dr. David Egan, UMC Utrecht, Utrecht, NL), used for baculovirus production, were cultured in Insect-XPRESS medium (Lonza, Basel, Switzerland, cat. No. BE12-730Q) supplemented with 5% FBS and pen/strep. Cultures were maintained at 27°C at 110 rpm.

### Cloning

sgRNAs targeting *AURKB*, *INCENP*, *CDCA8* and *BIRC5* were designed using the online CRISPR design tool at http://crispr.mit.edu/. The selected sgRNA sequences are listed in Table 1. The sgRNA and SpCas9 expression cassette from PX458 (pSpCas9(BB)-2A-GFP) or PX459 (pSpCas9(BB)-2A-Puro) (both plasmids were a gift from Dr. F. Zhang; Addgene, plasmids: # 48138 and # 62988 [7]) were cloned into the baculovirus donor plasmid pAceBac1 (a kind gift from Dr. Imre Berger, EMBL, Grenoble, France [76, 77]) from which the *polh* promoter was removed. Where applicable, the HDR template was introduced into a unique NotI site of the same donor vector. The HDR template for creating Aurora B H250Y consists of a 312 bp sequence ranging from chr17:8205171–8205482 (GRCh38) containing a mutation encoding H250Y and two additional silent mutations to prevent recognition by the sgRNA.

**Table 1:** List of sgRNA sequences used in this study.

purpose	sgRNA sequence
*AURKB* knockout	GCGCAGAGAGATCGAAATCC
*CDCA8* knockout	TAGGAAGGGCAGTAGTCGGG
*INCENP* knockout	CATGGAGTTTCTCTGCAACA
*BIRC5* knockout	CCAGGCAGGGGGCAACGTCG
*LUCIFERASE* control	GTCATTATAAATGTCGTTCGC
Introduction Aurora B H250Y mutation	GGGGCGCATGCACAATGAGA
Endogenous tagging of Haspin	CTTACTTAAACAGACTGTGC

For tagging of Haspin, two homology arms (HA), corresponding to chr17:3,726,333–3,727,360 (3’ RHA) and chr17:3,725,196–3,726,329 (5’ LHA), were cloned into pUNKI-20-C (Fig 5A). This yields a HDR template resulting in final integration of YFP at the 3’ end of Haspin, followed by an SV40 poly-A signal and a puromycin resistance cassette. The 5’ HA contains a silent mutation that removes the PAM site to prevent cutting by Cas9. The HDR template was then subcloned via NotI into the baculoviral Cas9-2A-GFP vector, containing the appropriate sgRNA (Table 1). Bacmids were generated using the Bac-to-Bac system in conjunction with EMBacY cells (a kind gift from Dr. Imre Berger, EMBL, Grenoble, France [76, 77]).

### Baculovirus production

Baculovirus was produced by transfection of bacmids into Sf9 cells. Cells were seeded in 6 well plates (0.5*10^6 cells/well), followed by transfection using FuGENE HD (Promega, Madison, WI, cat. no. E2311). P1 virus was harvested after 3–4 days and subsequently used to transduce 20 ml suspension cultures of early log phase Sf9 cells (1–2*10^6 cells/ml). P2 virus was harvested 3–4 days after transduction. Viral titers were determined using an end point dilution assay.

### Viral transduction

For baculoviral transduction, cells were trypsinized and taken up in RPMI-1640 (Sigma-Aldrich, St. Louis, MO, cat. no. R0883) supplemented with 10% heat inactivated FBS, 2 mM UltraGlutamine 100 units/ml penicillin and 100 μg/ml streptomycin, hereafter called RPMI-1640. The cells were then pelleted (5 min. at 483 x g) and re-suspended to a concentration of 75.000–200.000 cells/ml in RPMI-1640. Virus was added to the cell suspension at an MOI of 25–75 and cells were subsequently plated. For RPE-1 and MCF10A cells, the medium was changed back to their normal culture medium after 16 hours.

### Detection of indels

Cells were transduced as described above and after 2 days genomic DNA was isolated using a DNeasy Blood & Tissue Kit (Qiagen, Hilden, Germany). PCR was performed to amplify the sgRNA target locus. PCR products were run on agarose gel, purified using the QIAquick Gel Extraction Kit (Qiagen, Hilden, Germany) and sequenced using Sanger sequencing. Sequence traces were further analyzed using TIDE through the web interface at https://tide-calculator.nki.nl [30].

### Flow cytometry

To measure Cas9-2A-GFP expression, cells were transduced as described. After 14–16 hours the cells were trypsinized and taken up in PBS supplemented with 5% FBS and measured on a FACS-Calibur (Becton Dickinson, Breda, The Netherlands) using Cell Quest software (Becton Dickinson Breda, The Netherlands).

For the analysis of DNA content, cells were transduced with Cas9-2A-puro virus as described above. The next day 2 μg/ml puromycin (Sigma-Aldrich, St. Louis, MO, cat. no. P8833) was added for 24 hours. Four days after plating, cells were harvested and fixed with 70% ice-cold ethanol. Cells were washed with PBS containing 0.05% Tween20 (PBST) and finally taken up in PBS containing 250 ng/μl RNAse and 10 ng/μl propidium iodide. Samples were incubated at 37°C for 30 minutes and then measured on a FACS-Calibur using Cell Quest software.

### Immunofluorescence microscopy

Cells were transduced as described above and plated on 12 mm coverslips. Forty hours after cell plating, 20 μM S-Trityl-L-cysteine (STLC) (Tocris Bioscience, Bristol, UK, cat. no 2191) and 5 μM MG-132 (Merck Millipore, Billerica, MA, cat. no. 474790) were added to block cells in mitosis. Cells were fixed 2 hours later with 4% paraformaldehyde (PFA) in PBS for 7 minutes, and subsequently permeabilized with ice-cold methanol.

For the chromosome spreads, cells were transduced with baculoviruses carrying LacI-tagRFP or LacI-tagRFP-Pds5B as described above. Cells were blocked in mitosis by the addition of STLC (20 μM), 6 hours after plating. After another 14 hours nocodazol (0.83 μM) (Sigma-Aldrich, St. Louis, MO, cat. no. M1404) was added for 15 minutes, after which the cells were collected by mitotic shake-off. Cells were incubated in 75 mM KCl containing 0.1 μM okadaic acid (Merck Millipore, Billerica, MA, cat. no. 495604) at 37°C for 10 minutes and then transferred to cytocentrifuge chambers and spun onto coverslips using a Shandon Cytospin 4 for 5 minutes at 1500 rpm. For H3T3ph staining cells were fixed with 4% PFA in PBS for 7 minutes, and subsequently permeabilized with ice-cold methanol. For GFP staining, coverslips were incubated in 0.2% Triton X-100 in 100 mM PIPES pH6.8, 10 mM EGTA and 1 mM MgCl2 for 30–60 seconds, after which an equal amount of 4% PFA in PBS was added and incubated for 4 minutes (final concentration of 2% PFA). The fixative was replaced with 4% PFA in PBS and incubated for another 4 minutes.

Cells were then washed with PBS and blocked in PBST with 3% BSA. Coverslips were incubated with primary antibodies diluted in PBST with 3% BSA for 2 hours, washed three times with PBST, followed by a 1 hour incubation with the secondary antibodies in PBST with 3% BSA. DNA was then stained using 500 ng/ml DAPI in PBST followed by a final wash using PBS before mounting them onto glass slides using ProLong Gold Antifade Mountant (Thermo Fisher Scientific, Waltham, MA, cat. no. P36930). Primary antibodies used were anti-AIM1 (BD Biosciences, San Jose, CA, cat. no. 611083, mouse, monoclonal, 1:500), anti-CENP-C (MBL International, Woburn, MA, cat. no. PD030, guinea pig, polyclonal, 1:1000), anti-phospho-Histone H3 (Ser10) (Merck Millipore, Billerica, MA, cat. no. 06–570, rabbit, polyclonal, 1:1000), anti-phospho-Histone H3 (Thr3) (Merck Millipore, Billerica, MA, cat. no. 07–424, rabbit, polyclonal, 1:2000), anti-RFP (Chromotek, Planegg-Martinsried, Germany, cat. no. 5F8, rat, monoclonal, 1:1000), and anti-GFP (a kind gift from Dr. G.J.P.L. Kops, rabbit, polyclonal, 1:1000). Secondary antibodies used were goat anti-mouse IgG Alexa Fluor 568 conjugate (Thermo Fisher Scientific, Waltham, MA, cat. no. A-11031, 1:600), goat anti-rabbit IgG Alexa Fluor 488 conjugate (Thermo Fisher Scientific, Waltham, MA, cat. no. A-11034, 1:600), goat anti-rat IgG Alexa Fluor 568 conjugate (Thermo Fisher Scientific, Waltham, MA, cat. no. A-11077, 1:600), and goat anti-guinea pig IgG Alexa Fluor 647 conjugate (Thermo Fisher Scientific, Waltham, MA, cat. no. A-21450, 1:600).

Images were acquired on a DeltaVision-RT imaging system (GE Healthcare, Chicago, Il), upgraded with a 7 color InsightSSI Module & TruLight Illumination System Module using a UPlanSApo 100x/1,40 objective and a CoolSnap HQ2 camera (Photometrics). Presented images are deconvolved maximum intensity projections, processed using Softworx v6. Quantifications were performed using ImageJ. The percentage of knockout cells was scored by eye on a Zeiss AxioImager Z1 using the 63x PLAN-ApoChromat objective.

### Live cell imaging

Cells were transduced as described above, and then re-plated 14–16 hours after transduction in 24 well-plates (Sigma-Aldrich, St. Louis, MO, cat. no. CLS3524). Thymidine (Sigma-Aldrich, St. Louis, MO, cat. no. T1895) was added to a final concentration of 2.5 mM. After 24 hours cells were released from the thymidine block by washing 3x with medium, followed by the addition of Leibovitz’s L-15 medium (Thermo Fisher Scientific, Waltham, MA, cat. no. 21083027) supplemented with 6% FBS, 2 mM UltraGlutamine, 100 units/ml penicillin and 100 μg/ml streptomycin, hereafter called Leibovitz’s L-15. Cells were imaged on an Olympus Cell M system with a UPlanFL N 20x/0.5 objective and analyzed using Cell M software. For the paclitaxel override experiments, cells were plated in 24-well plates as described above in the presence of 2.5 mM thymidine and 2 μM ZM447439 (Tocris Bioscience, Bristol, UK, cat. no. 2458) or DMSO. After 24 hours cells were washed 3 times with medium to remove thymidine, followed by the addition of medium containing 1 μM paclitaxel (Sigma-Aldrich, St. Louis, MO, cat. no. T1912) and 2 μM ZM447439 or DMSO. After ~4.5 hours, medium was changed to Leibovitz’s L-15 medium containing 1 μM paclitaxel and 2 μM ZM447439 or DMSO. Cells were imaged on an Olympus Cell M system as described above.

U-2 OS-LacO Haspin-YFP or RPE-1 Haspin-YFP cells and untagged parental controls were plated in a μ-slide four well glass bottom dish in FluoroBrite DMEM supplemented with 10% FBS (IBIDI, cat. no. 80427). Cells were then blocked in G2 for 16 hours with the Cdk1 inhibitor RO-3036 (7.5 μM). DNA was then stained by the addition of Hoechst 33342 (5 μM) for 30 minutes, followed by a release of the RO-3036 block (washing three times) in FluoroBrite DMEM supplemented with 10% FBS. After approximately 30 minutes images were acquired at 37°C on a DeltaVision imaging system (GE Healthcare, Chicago, Il), upgraded with a 7 color InsightSSI Module & TruLight Illumination System Module using a PlanApo N 60x/1,42 objective and a CoolSnap HQ2 camera (Photometrics) with 3x3 binning. Displayed images are single Z-stacks.

### Western blotting

For analysis of Cas9 expression, cells were harvested 14–16 hours after transduction. For examination of the CPC components, cells were transduced, followed after 24 hours, by treatment with the Cdk1 inhibitor RO-3306 (7.5 μM) (Merck Millipore, Billerica, MA, cat. no. 217699). After 14–16 hours, cells were released from the RO-3306 block by washing 3 times with warm medium, followed by the addition of MG-132 (5 μM) after 30 minutes. Mitotic cells were harvested by shake-off, 90 minutes after addition of MG132. Protein concentration was determined by Lowry assay to ensure equal sample loading. Proteins were separated by SDS-PAGE using a 7.5% polyacrylamide gel for Cas9 expression or a Bolt 4–12% Bis-Tris Plus gel for expression of the CPC components (Thermo Fisher Scientific, Waltham, MA, cat. no. NW0412BOX). Proteins were then transferred to nitrocellulose membranes using a Trans-Blot Turbo Transfer system (Bio-Rad, Hercules, CA, cat. no. 170–4155). Membranes were blocked in PBST containing 3% BSA. Primary antibody incubation was performed in PBST including 3% BSA for ~18 hours at 4°C. Membranes were washed three times with PBST before incubation with the secondary antibody diluted in PBST. Membranes were washed three times with PBS prior to image acquisition on an ImageQuant LAS4000 (GE Healthcare, Chicago, Il). Signals were quantified using ImageJ. Primary antibodies used were anti-AIM1 (BD Biosciences, San Jose, CA, cat. no. 611083, mouse, monoclonal, 1:250), anti-Borealin (a generous gift from Dr. S.P. Wheatley, rabbit, polyclonal, 1:10,000), anti-INCENP (Thermo Fisher Scientific, Waltham, MA, cat. no. 39–2800, mouse, monoclonal, 1:500), anti-Survivin (R&D Systems, Minneapolis, MN, cat. no. AF886, rabbit, polyclonal, 1:10,000), anti-α-tubulin (Sigma-Aldrich, St. Louis, MO, cat. no. T5168, mouse, monoclonal, 1:10,000), and anti-Cas9 (Diagenode, Liège, Belgium, cat. no. C15200203, mouse, monoclonal, 1:1000). Secondary antibodies used were ECL Plex Goat-α-Rabbit IgG-Cy3 (GE Healthcare, Chicago, Il, cat. no. 28-9011-06, 1:2500) and ECL Plex Goat-α-Mouse IgG-Cy5 (GE Healthcare, Chicago, Il, cat. no. PA45009, 1:2500).

### Generation of cell lines with Aurora B H250Y mutation

Cells were transduced with the appropriate virus as described above. After ~ 2 hours, SCR7 (ApexBio, Houston, TX, cat. no. A8705) was added to a final concentration of 1 μM. 18–20 hours after plating the cells the medium was replaced with medium containing 2 μg/ml puromycin for 24 hours. After recovery from selection, cells were replated in 0.4 μM ZM447439. Single clones were picked and expanded. In order to determine the genotype of individual clones, genomic DNA was isolated (DNeasy Blood & Tissue Kit; Qiagen, Hilden, Germany). PCR was performed to amplify the target loci. PCR products were purified using the QIAquick PCR Purification Kit (Qiagen, Hilden, Germany) and sequenced using Sanger sequencing.

### Generation of Haspin-YFP cell lines

Cells were transduced with the appropriate virus as described above. For RPE-1 cells the medium was refreshed after 24 hours, followed by the addition of puromycin after another 24 hours to a final concentration 10 μg/ml. Individual clones were then allowed to grow out and subsequently picked, expanded and tested for Haspin-YFP expression using fluorescence microscopy. For U-2 OS-LacO cells the medium was refreshed after 24 hours. After another 24 hours the cells were harvested and subsequently seeded into 96 well plates at a density of approximately 30 cells per plate. Cells were grown in the presence of puromycin (2.5 μg/ml). Clones that grew out were tested using fluorescence microscopy. All clones that tested positive for fluorescence were then further characterized at the genomic level. Genomic DNA was isolated (DNeasy Blood & Tissue Kit; Qiagen, Hilden, Germany) and correct integration of the HDR template was confirmed through both PCR and Sanger sequencing.

### Colony formation assay

2000 cells per well were plated in 6-well plates. After 24 hours, medium with 0.4 μM ZM447439 or 2 μg/ml puromycin was added as indicated. Cells were fixed ~7 days after addition of the inhibitors using 4% PFA in PBS for 15 minutes. Cells were subsequently stained using 0.2% crystal violet for 10 minutes.

## Supporting information

S1 Fig Transduction of U-2 OS cells with CRISPR/Cas9 baculoviruses leads to gene disruption. A) Representative FACS-profiles showing GFP expression in U-2 OS cells treated with the indicated CRISPR/Cas9 baculoviruses (MOI 25). The markers are set such that 2% of the cells treated with Cas9-2A-puro baculovirus are included in this region. The percentage of cells treated with Cas9-2A-GFP baculoviruses within the marker region is indicated. B) Uncropped Western blot showing expression of Cas9 in U-2 OS cells treated with CRISPR/Cas9 baculoviruses (MOI: 25). α-tubulin was used as a loading control. The Western blot corresponds to the cropped images in Fig 1C. C) Sequencing chromatograms of the Aurora B, Borealin, INCENP, and Survivin sgRNA target loci without treatment or after treatment with the indicated CRISPR/Cas9 baculoviruses (MOI: 25). PAM sites are indicated and the arrows point out the predicted CRISPR/Cas9 cleavage sites. The corresponding amino acids are shown in the chromatograms of the untreated cells. Note that the target site of Aurora B was chosen further downstream in the gene to ensure disruption of all known Aurora B isoforms. The percentage of indels and corresponding R^2^ as determined by TIDE analysis is shown.
(PDF)

S2 Fig Western blot of CPC members from mitotic U-2 OS cells treated with CRISPR/Cas9 baculoviruses (MOI: 25). α-tubulin was used as a loading control. The Western blot corresponds to the cropped images in Fig 2A.
(PDF)

S3 Fig Representative live cell images of U-2 OS-LacO cells and RPE-1 cells in prometaphase, anaphase and interphase. These cell lines are the parental controls for the cells with endogenously tagged Haspin (Fig 5C).
(PDF)
