# Supplementary figures and images for: Baculoviral delivery of CRISPR/Cas9 facilitates efficient genome editing in human cells
Source: PLoS One. 2017 Jun 22;12(6):e0179514. doi: 10.1371/journal.pone.0179514 (PMC5480884; doi:10.1371/journal.pone.0179514)

Supplemental figure 1

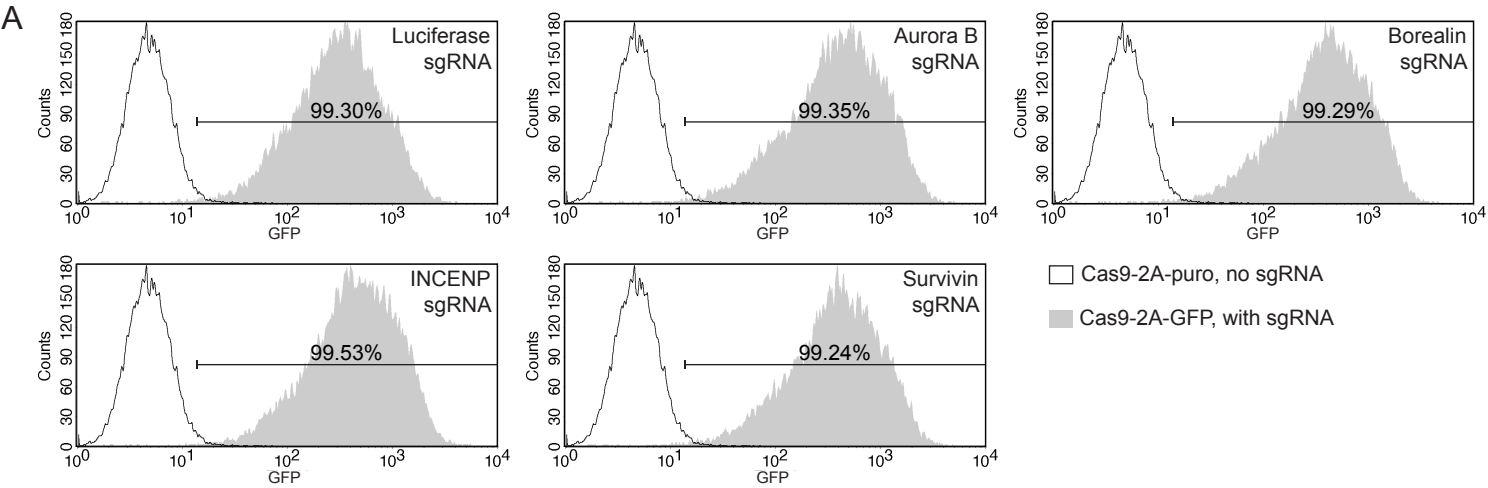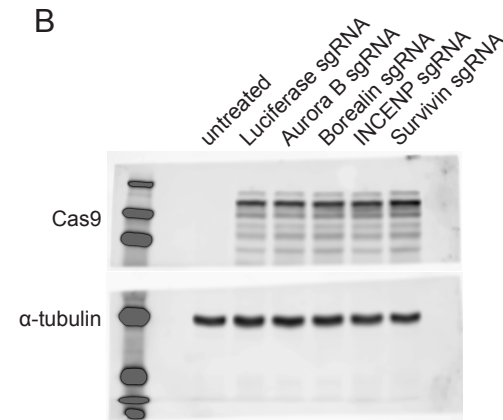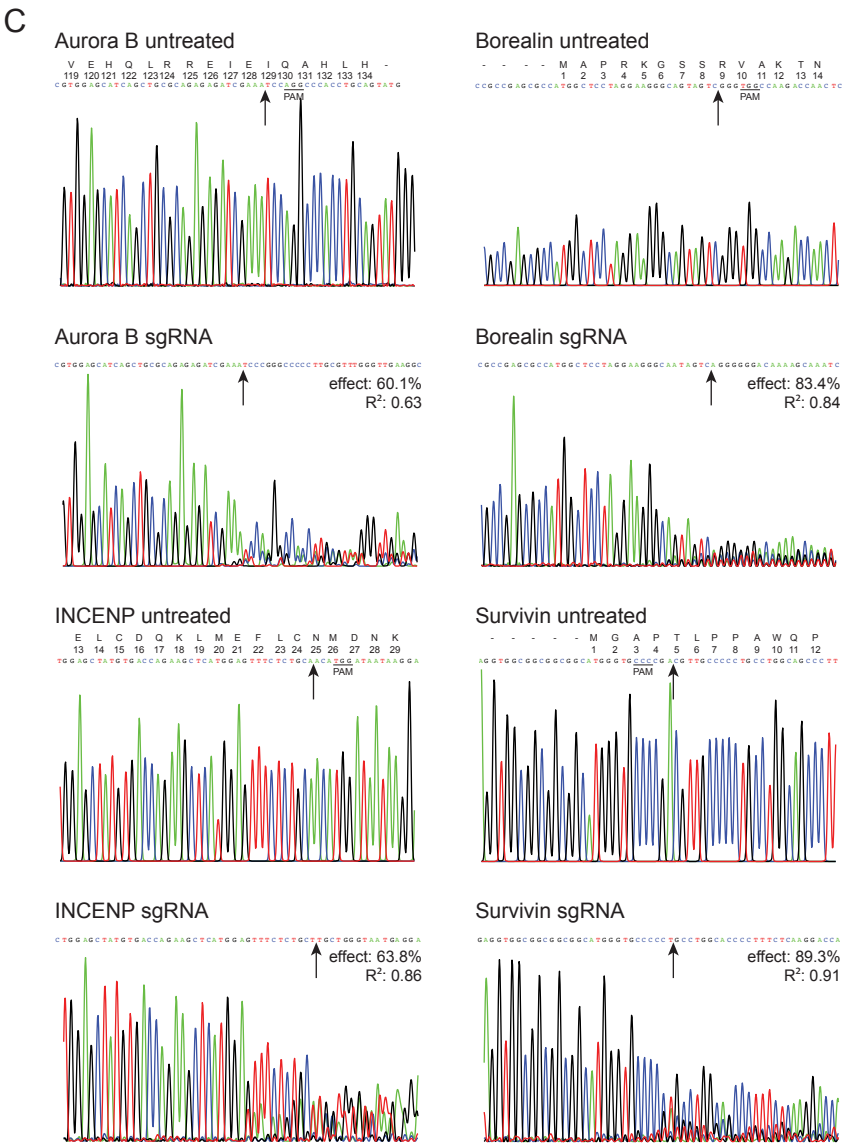

Supplement: S1 Fig — A) Representative FACS-profiles showing GFP expression in U-2 OS cells treated with the indicated CRISPR/Cas9 baculoviruses (MOI 25). The markers are set such that 2% of the cells treated with Cas9-2A-puro baculovirus are included in this region. The percentage of cells treated with Cas9-2A-GFP baculoviruses within the marker region is indicated. B) Uncropped Western blot showing expression of Cas9 in U-2 OS cells treated with CRISPR/Cas9 baculoviruses (MOI: 25). α-tubulin was used as a loading control. The Western blot corresponds to the cropped images in Fig 1C. C) Sequencing chromatograms of the Aurora B, Borealin, INCENP, and Survivin sgRNA target loci without treatment or after treatment with the indicated CRISPR/Cas9 baculoviruses (MOI: 25). PAM sites are indicated and the arrows point out the predicted CRISPR/Cas9 cleavage sites. The corresponding amino acids are shown in the chromatograms of the untreated cells. Note that the target site of Aurora B was chosen further downstream in the gene to ensure disruption of all known Aurora B isoforms. The percentage of indels and corresponding R2 as determined by TIDE analysis is shown. (PDF) [file pone.0179514.s001.pdf]

Supplemental figure 2

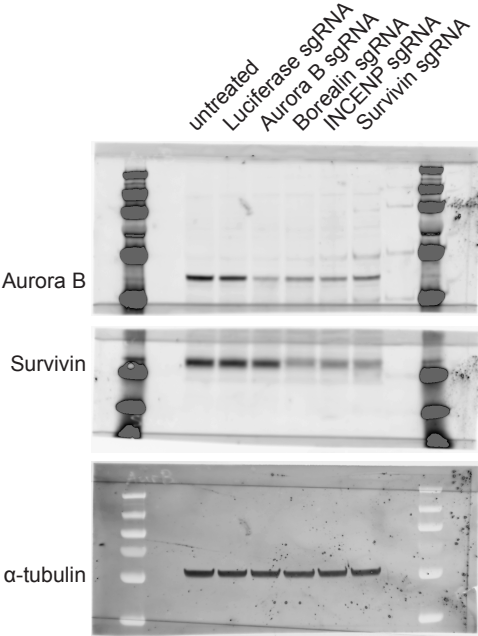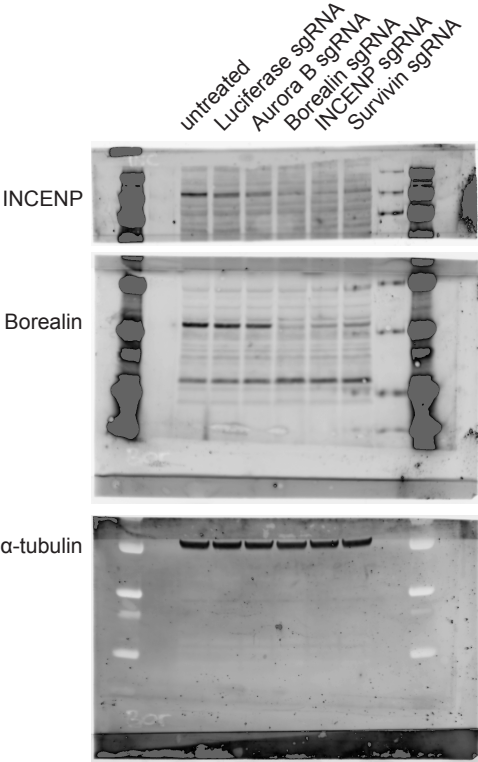

Supplement: S2 Fig — α-tubulin was used as a loading control. The Western blot corresponds to the cropped images in Fig 2A. (PDF) [file pone.0179514.s002.pdf]

Supplemental figure 3

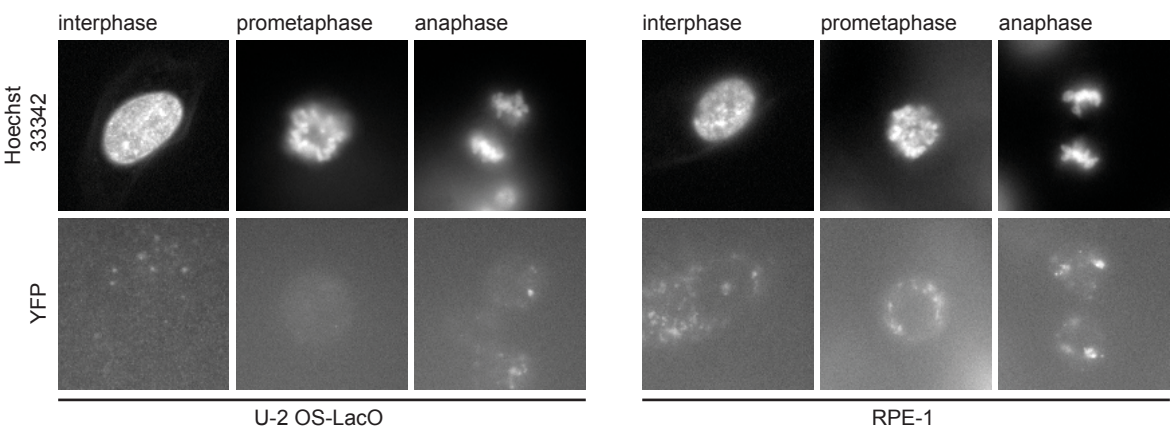

Supplement: S3 Fig — These cell lines are the parental controls for the cells with endogenously tagged Haspin (Fig 5C). (PDF) [file pone.0179514.s003.pdf]
